# Dynamic responses of a vibro-impact capsule robot self-propelling in the large intestine via multibody dynamics

**DOI:** 10.1007/s11044-025-10052-6

**Published:** 2025-02-04

**Authors:** Zepeng Wang, Jiyuan Tian, Yang Liu, Ana Neves, Shyam Prasad

**Affiliations:** 1 Exeter Small-Scale Robotics Laboratory, Engineering Department, University of Exeter, Exeter, EX4 4QF UK; 2 Engineering Department, University of Exeter, Exeter, EX4 4QF UK; 3 Royal Devon and Exeter NHS Foundation Trust, Barrack Road, Exeter, EX2 5DW UK

**Keywords:** Vibro-impact, Capsule robot, Multibody dynamics, MSC Adams, Experiment

## Abstract

In recent years, colonic capsule endoscopy has become available in clinical practice as an alternative modality to colonoscopy. However, it faces challenges such as prolonged examination time and the absence of clinician navigation. Leveraging their pioneering work in the field of vibro-impact self-propulsion technique for gastrointestinal endoscopy, Zhang et al. (IEEE Robot. Autom. Lett. 8:1842–1849, 2023) developed a novel, untethered, self-propelled, endoscopic capsule robot, with the aim of providing a new means of examining bowel cancer in real time. To evaluate and optimize the passage of this capsule robot self-propelling in the large intestine, this work adopts multibody dynamics analysis and experimental investigation to study the robot’s dynamics and its interaction with the intestinal environment. Considering the complex anatomy of the large intestine, containing different sections, e.g., cecum, ascending, transverse, descending, and sigmoid colon, and variations of the haustra, e.g., with various radii, lengths, and heights, the robot was driven by the square-wave excitation of an inner mass interacting with the capsule body and tested on a real porcine colon. The robot’s driving parameters, including the excitation frequency, amplitude, and duty cycle, and the dimensions of the haustra are the two main factors influencing the robot’s progression in the intestine. By comparing with the experimental results, the proposed multibody dynamics model developed using MSC Adams can estimate the movement of the capsule robot and the intestinal resistance quantitatively. Extensive numerical and experimental studies suggest an excitation frequency of 60 Hz and a duty cycle of 0.4 as the optimal parameters for driving the robot, and the longer the haustral length is, the faster the robot passes through. These results ensure the validity of the proposed multibody dynamics platform, which can be used by robotic engineers for developing medical robots for intestinal examinations.

**Supplementary Information:**

The online version contains supplementary material available at 10.1007/s11044-025-10052-6.

## Introduction

Colorectal cancer, the third most common cancer and one of the leading causes of cancer-related deaths worldwide, highlights the urgent need for early detection [2, 3]. Various methods have been developed for the diagnosis of colorectal cancer, including the detection of DNA markers, serum marker screening, and microscopy see e.g. [4–7]. Among them, colonoscopy is considered the gold standard for colorectal cancer screening and early diagnosis [8, 9]. Unfortunately, this has limited the development of colonoscopy due to the risks associated with invasive colonoscopy, such as discomfort and potential intestinal bleeding [10, 11]. As a result, researchers are working to find safer and more comfortable alternative screening methods.

Capsule endoscope is a non-invasive procedure in which the patient simply swallows a capsule equipped with a miniturized camera without inserting an endoscope [12], reducing discomfort and risk [13]. It can explore parts of the body that cannot be directly visualized via conventional endoscopy, such as the small intestine [14]. However, passive capsules have the drawback of lengthy examination time, approximately 10 hours, and limited diagnostic coverage due to the lack of clinician’s navigation, which may result in potentially missed abnormalities, see e.g. [15–17].

**Fig. 1:**
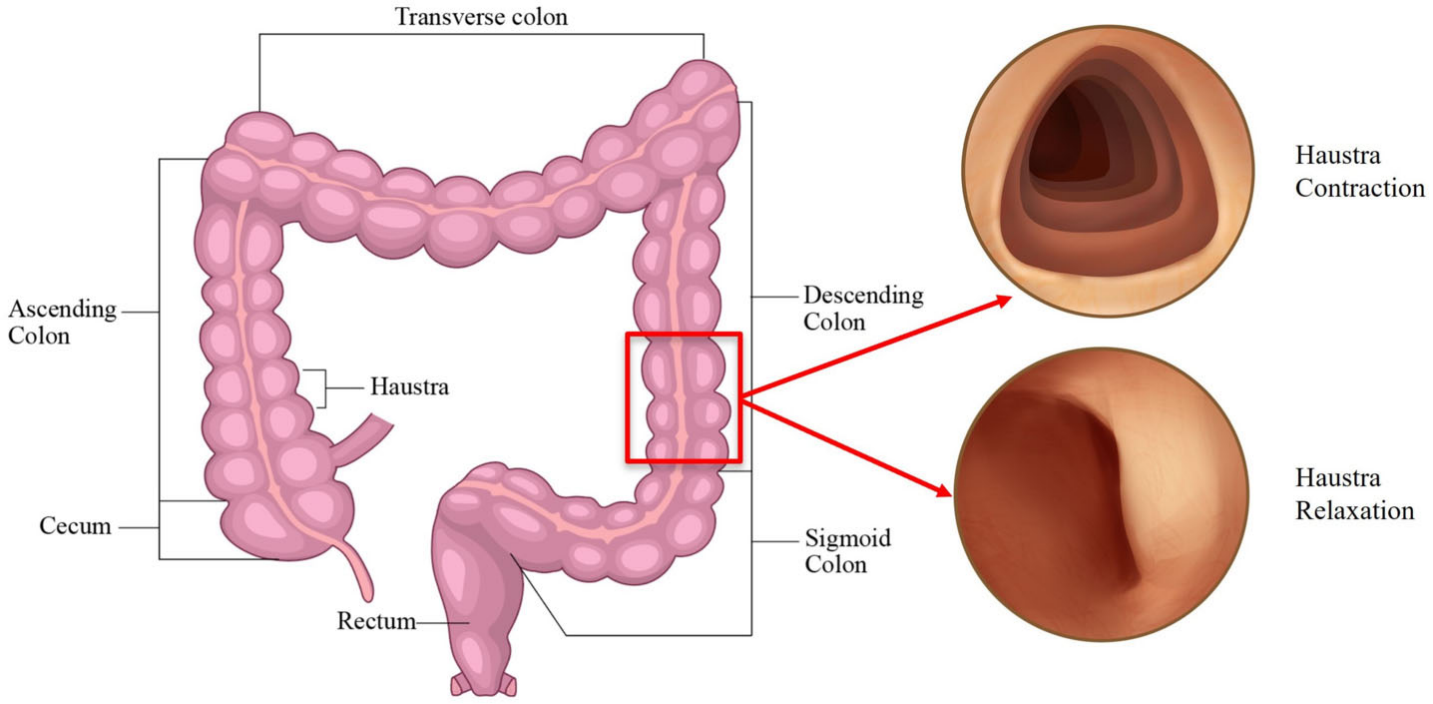
The anatomy and the cross-sectional view of various sections of the human colon. The colon, characterized by its tubular and winding structure, has distinct folds and measures approximately 1.5 to 2.0 meters in length. It has a larger diameter compared to the small intestine and is composed of multiple haustra. The colon is shorter than the small intestine and consists of six sections: rectum, sigmoid colon, descending colon, transverse colon, ascending colon, and cecum. The ascending colon, the descending colon, and the rectum are located behind the peritoneum and are fixed within the abdominal cavity

Addressing the control of capsule movement within the intestines is a highly challenging and critical task. After ingesting by the patient, the capsule must navigate through the stomach, the small intestine, and the colon [18]. The colon, with its tubular and winding structure, has distinct folds and is about 1.5–2.0 meters long with a larger diameter, composed of multiple haustra [19]. Compared to the small intestine, the colon is shorter and consists of six sections as illustrated in Fig. 1: the rectum, sigmoid colon, descending colon, transverse colon, ascending colon, and cecum [20]. The ascending colon, descending colon, and rectum are located behind the peritoneum and are fixed within the abdominal cavity [21]. However, the colon’s unique convoluted shape and haustra-like structure present high protrusions and sharp turns at the junctions of its parts, posing a significant challenge for navigating the capsule through curved intestinal paths, climbing over, and traversing the ridges. Researchers have proposed using anatomy and physiology to investigate the dynamic motion patterns of the intestine, specifically peristalsis and segmental contraction [22]. They have transformed these patterns into virtual and physical test simulators to evaluate the performance of the intestinal model [23]. For instance, Bertuzzi et al. [24] proposed simulating peristalsis through the stationarity and deformation of intestinal units. Miftakhov explored the deformation and contraction velocity of the intestinal wall and established a model of intestinal peristalsis [25]. As the self-propelled capsule robot has emerged as a promising diagnostic method for examining the digestive tract [26, 27], numerical simulations and experimental studies have been conducted on the interaction between the robot and the intestine to study the factors, such as the contact pressure [28, 29], the intestinal friction [30–33], and the movement speed of the robot [34, 35]. Recent studies have modeled the gut as a viscoelastic material due to its stress relaxation properties. However, previous research works primarily focused on the movement of the capsule in a simplified straight intestine through finite element analysis, with very few utilizing multibody dynamics (MBD) analysis to simulate the anatomy of the intestine, which is critical for understanding the capsule’s dynamics in the intestinal environment. Therefore, this work aims to study the dynamics of the capsule robot in a more realistic large intestinal environment by employing MBD analysis, which has not been studied before.

Since the introduction of capsule endoscopy in 2001, the wireless capsule endoscope for colon examination has garnered significant attention from researchers [36]. Reports indicate that this type of endoscope has an effective examination rate exceeding 90% without associated complications [37]. Despite the commercialization of several capsule endoscopes, they are primarily propelled by intestinal movements [38–40]. This passive propulsion leads to several drawbacks, including an examination duration typically around 10 hours, unpredictable and uncontrollable movement speeds, poor visibility leading to missed lesions, and a certain rate of retention [41]. These issues result in low usage rates of capsule endoscopes in medical examinations and low acceptance among patients. To tackle these challenges, researchers have made significant progresses towards enhancing the capsule’s controllability for gastrointestinal diagnostics [42]. A diverse array of active propulsion mechanisms have been developed to improve the capsule’s autonomy, such as the spring-loaded crawling systems utilizing memory alloys [43], caterpillar-like locomotive modules [44], and innovative designs resembling inchworm structures [45]. Additionally, the propeller-based capsule [46] and the soft snail-like robot [47] offer alternative avenues. It is worth noting that among these is the growing field of magnetically-guided actuation methods, comprising the analog magnetic actuation [48], the positioning endoscopy system [49], the commercially available magnetically controlled gastric capsule [50], and the levitation system engineered to minimize the environmental contact and facilitate smoother motion [51].

**Fig. 2:**
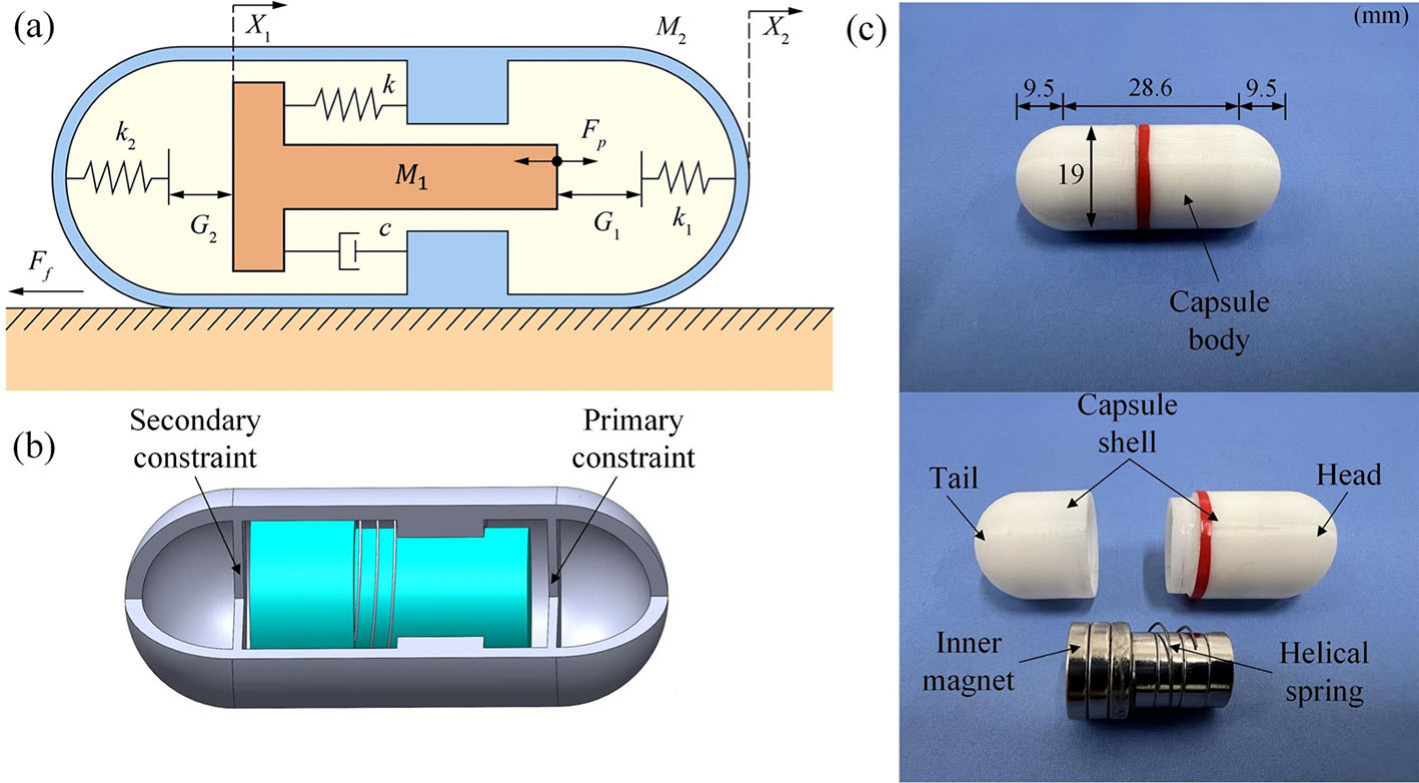
(a) Physical model of the vibro-impact capsule robot, where $k_{1}$, $k_{2}$, $G_{1}$, $G_{2}$ represent the stiffness coefficients of the primary and secondary constraint and their clearances with the inner mass block, respectively. $k$ and $c$ are the stiffness and damping coefficients of the helical spring for reverting the mass block. $M_{1}$, $M_{2}$, $X_{1}$, $X_{2}$ are the mass and the displacement of the inner mass and the capsule body, respectively. (b) Internal structure of the vibro-impact capsule robot. (c) Photographs of the assembled and disassembled capsule robot, indicating a total length of 47.6 mm and an outer diameter of 19 mm, consists of a capsule tail shell, a head shell, an inner permanent magnet, and a helical linear spring

The development of magnetically-guided driving methods for capsule endoscope holds significant importance owing to its inherent advantages, notably the avoidance of direct patient contact and enhanced precision. Based on their previous work [52], Zhang et al. [1] developed a capsule robot consisting of a vibro-impact system for self-propulsion. As illustrated in Fig. 2(a), the robot comprises a primary and a secondary constraint characterized by stiffness coefficients $k_{1}$ and $k_{2}$, respectively. Connecting the inner mass ($M_{1}$) to the capsule body ($M_{2}$) is a helical spring with stiffness $k$ and damping coefficient $c$. Here, $F_{p}$ represents the periodic force acting on the inner mass, while $X_{1}$ and $X_{2}$ denote the absolute displacements of the mass block and the shell, respectively. $G_{1}$ and $G_{2}$ represent the gaps between the mass block and the primary and the secondary constraints, respectively. When the absolute displacement difference $X_{1}-X_{2}$ equals or exceeds these gaps, the mass block collides with either the primary or the secondary constraint, exerting an impact force on the capsule body. Subsequently, if the net force on the capsule body surpasses the intestinal resistance ($F_{f}$), the capsule moves either forward or backward. Prior research has demonstrated that during the resonance of the capsule robot, it can achieve its maximal progression speed, effectively overcoming the haustral folds and navigating through tight junctions [53]. However, these existing works only considered the capsule robot moving in a simplified environment, without accounting for additional dimensions. Thus, the present work aims to fill this gap by studying the robot’s dynamics in a complex 3D environment via MBD analysis.

Previous studies, involving numerical simulations of the capsule robot, have focused on the interaction dynamics between the capsule and the intestine, highlighting the critical role of contact condition and resistance in simulating the capsule’s movement within the intestinal environment. Notably, Zhang et al. [1] demonstrated the feasibility of the vibro-impact capsule being navigated through the complete and natural terrain of the large intestine. However, the unique anatomical and physiological complexities of the large intestine pose specific challenges, see e.g. [20], which requires a deeper comprehension of the capsule’s navigation within this region. Given the intricate structural features of the large intestine, such as varying the dimensions of the haustra, folds, and flexures, the direction and conditions of the capsule’s movement remain dynamic and pose hurdles in achieving precise navigation. Nonetheless, employing MBD holds promise in accurately capturing and simulating these intricate movements and their implications for the capsule’s motion. Therefore, MBD can provide a platform for comparing simulation outcomes with real-world experiments, enabling robotic engineers to refine their capsule designs and optimize their performance.

To comprehensively study the locomotion and the dynamical responses of the vibro-impact capsule robot within a realistic intestinal environment, the present work aims to develop an MBD model using MSC Adams (Automated Dynamic Analysis of Mechanical Systems) software and validate it through experiments. This study will explore the capsule’s dynamics by comparing the MBD model with the experimental prototype under various scenarios, including the intact large intestine, different flexures, and the haustra in various dimensions. As a continuation of the design adopted from [1], the contributions of this work are as follows. (i) An MBD visualization platform was designed for testing the self-propelled capsule robots. (ii) The study considered the movement of the robot under different driving parameters within a complete colon. (iii) It investigated the colons with varying bending angles. (iv) It examined the impact of different haustra dimensions on the capsule’s movement. While robotic researchers often focus on robot design and performance demonstration, our study approaches the problem from a multibody dynamics system perspective, placing emphasis on the intricate interactions between the capsule and the intestinal environment. The exploration of capsule-intestine interaction dynamics within the framework of MBD is a relatively underexplored area, making this work unique and novel. Furthermore, although the present work leverages MSC Adams for its MBD simulations, it contributes to the application of MBD methodologies in the context of medical robots, an emerging area of research.

The rest of this paper is organized as follows. Section 2 studies the MBD model of the capsule’s movement within the colon. Considering the complex anatomy of the colon, three different motion scenarios were considered: the complete colon, the colon segments with different bending angles, and the colon segments with different dimensions of the haustra. Furthermore, the parameters of the capsule prototype were identified, and the material descriptions of the colon and the capsule, as well as the configuration of the MBD model, were introduced. Section 3 introduces the experimental platform for robot testing, including a complete colon and several colon segments with different bending angles under various excitation parameters. Section 4 presents the results of the capsule’s movement in three scenarios, including the comparisons of the MBD simulations and the experimental results of the capsule’s movement in the large intestine and curved colon segments under different excitation parameters, and a kinematic analysis of the robot obtained through simulation. Finally, concluding remarks are drawn in Sect. 5. In addition, supplementary video is included to provide a comprehensive understanding of the experimental and simulation setups.

## Modeling and methods

In order to assess and refine the motion dynamics of the vibro-impact capsule within the large intestine, this study proposes to utilize the rigid and flexible coupling capabilities provided by MSC Adams 2020 software for MBD analysis. This approach will facilitate comprehensive modeling of the capsule-intestine interaction, taking into account both geometric structure and material properties. Key considerations include configuring contact settings, defining rigid and flexible body properties, optimizing meshing, parameterizing driving forces and helical springs, establishing boundary conditions, and applying relevant loads.

### Geometrical modeling and analysis strategy

In the design of the vibro-impact capsule, there are several key considerations that guide the process. Firstly, the capsule’s overall dimensions should facilitate easy movement and rotation within the large intestine. Secondly, the internal magnet’s volume should be large enough to generate the necessary driving force under the external magnetic field. Moreover, ensuring complete sealing of the capsules within the operational environment is crucial to prevent the ingress of intestinal fluid. In addition, the capsule’s overall shape should be considered continuous and without protruding accessories to minimize potential damage to the intestinal wall. Addressing these requirements, this study adopts a previously established capsule design structure, depicted in Figs. 2(b) and (c). This structure comprises the capsule shell, primary and secondary constraints, spiral springs, and inner mass for assembly. The capsule’s dimensions, determined by these requirements, are set at 47.6 mm in length and 19 mm in diameter. The inner mass consists of a T-shaped permanent magnet, stimulated by a periodic square wave signal characterized by specific amplitude (driving force), frequency, and duty cycle to propel the capsule motion. Subsequently, the inner magnet connected to the helical spring mainly experiences mutual collisions with the primary and secondary constraints, generating forward or backward motion on the capsule. In detail, the T-shaped magnet’s head has 15 mm in diameter and 9 mm in length, while its tail has a diameter of 12 mm and a length of 15 mm.

**Fig. 3:**
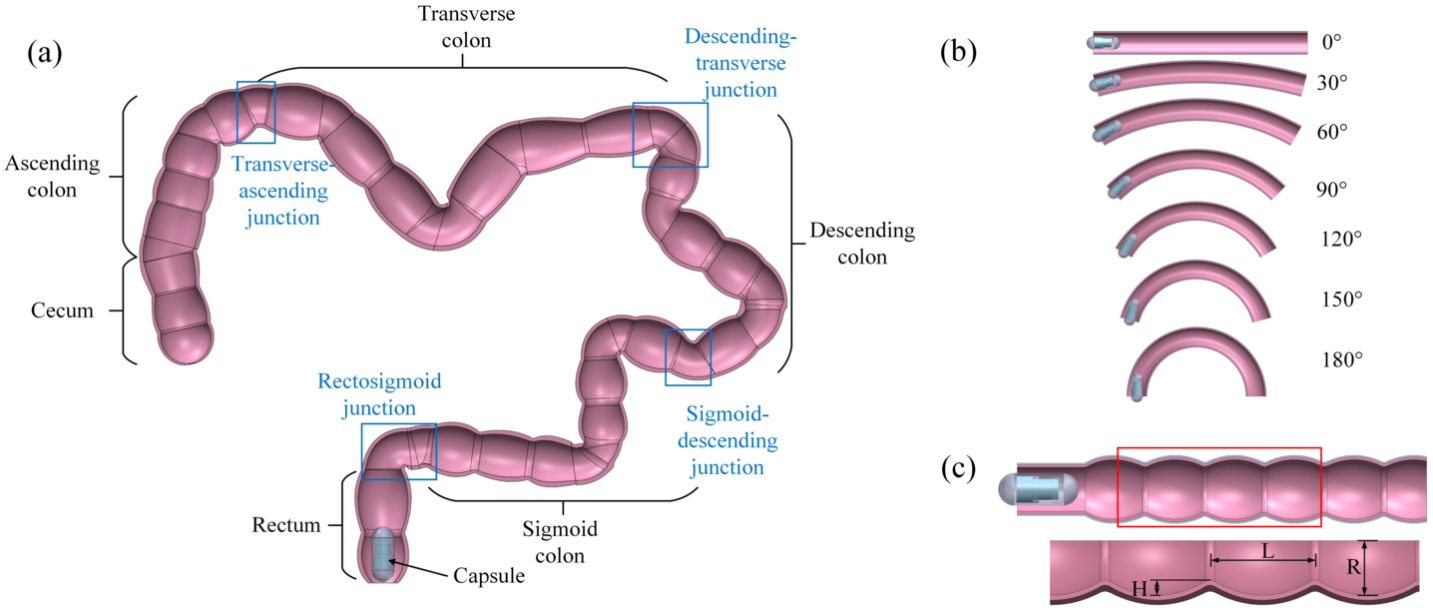
Different capsule motion scenarios considered: (a) Scenario 1: The capsule robot is driven in a complete large intestine/colon (as shown in pink) moving under different excitation parameters. The large intestine has a corresponding physiological structure of the human body with a total length of 1.6 m. (b) Scenario 2: The capsule moves through the colon segments with different bending radii ranging from 0^∘^ to 180^∘^ at a frequency of 60 Hz, 0.4 duty cycle, and an amplitude of 120 mN. (c) Scenario 3: The capsule moves in a straight colon segment with different intestinal radius $R$ and length $L$, and fold height $H$ under the control parameter of an amplitude of 120 mN, a frequency of 60 Hz, and a duty cycle of 0.4 (Color figure online)

Scenario 1 explores the optimal excitation parameters for capsule velocity within the large intestine, with a focus on assessing the agreement between simulation and experimental results. The large intestine model encompasses the anatomical structures of the rectum, sigmoid colon, descending colon, transverse colon, and ascending colon. Given the inherent differences in length, diameter, fold height, and the number of intestinal folds across these segments within the human body, dimensions were determined based on data provided by [20], resulting in a model length of approximately 1.6 meters. Furthermore, to simulate the physiological environment of the human large intestine, a porcine colon layer was used in the experimental setup. The porcine colon, highlighted in pink in Fig. 3(a), closely resembles the human colon in terms of mechanical properties and dimensions. It was prepared and mounted on a rigid holder to replicate the natural conditions for capsule movement studies. To further explore capsule mobility, as illustrated in Scenario 2 in Fig. 3(b), the intestinal segments were designed with different bending radii between 0^∘^ to 180^∘^ at intervals of 30^∘^, with each segment featuring a fixed diameter of 28 mm. Scenario 3 depicted in Fig. 3(c) investigates the impact of haustral size on capsule movement, specifically examining changes in radii $R$ from 17. 5 mm to 27.5 mm and fold heights $H$ from 2 mm to 6 mm. In addition, the haustral lengths $L$ varied from 20 mm to 50 mm at intervals of 5 mm. These parameters were determined based on average values sourced in [54, 55].

### Intestinal material descriptions

**Fig. 4:**
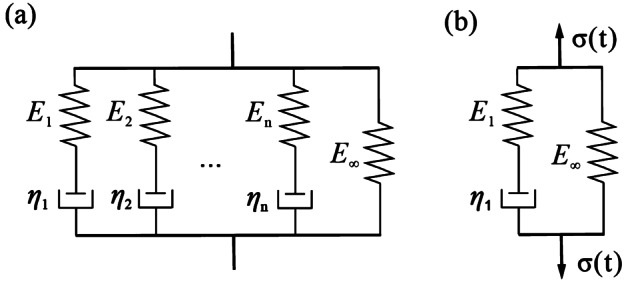
Viscoelastic material models of the intestine: (a) generalized Maxwell model (adopted from [30]) and (b) three-element Maxwell model (adopted from [27])

The distinctive elastic and viscous attributes of the small intestine emerge from the coordinated peristalsis of smooth muscle and the lubricating function of mucus secreted by intestinal glands. The generalized viscoelastic Maxwell model stands as a prevalent framework for elucidating its mechanical behavior. The model consists of a spring and multiple parallel spring-damper chains, as shown in Fig. 4(a). The model consists of several identical components arranged in parallel, with the chain length adjustable to the relaxation time of the material. Moreover, the relaxation modulus $(E) $ represents a key viscoelastic property, delineating how materials relieve stress over time $(t)$. Thus, in accordance with the generalized Maxwell model, $E $ can be formulated as

1 $$ E(t)=\sum _{i=1}^{n} E_{i} e^{-\frac{E_{i}}{\eta _{i}}}+E_{\infty}, $$

where $n$ is the number of spring and punch series, $\eta _{i}$ is the viscosity of the damper, $E_{i}$ is the elastic modulus of the spring, and $E_{\infty}$ is Young’s modulus of the model when $t$ is close to $\infty $. Drawing from previous experimental findings [27], a three-dimensional model as shown in Fig. 4(b), incorporating two elastic springs and a viscous damper arranged in series, presents a viable approach for simulating the viscoelastic characteristics of the intestine.

### Characterization of the helical spring

**Fig. 5:**
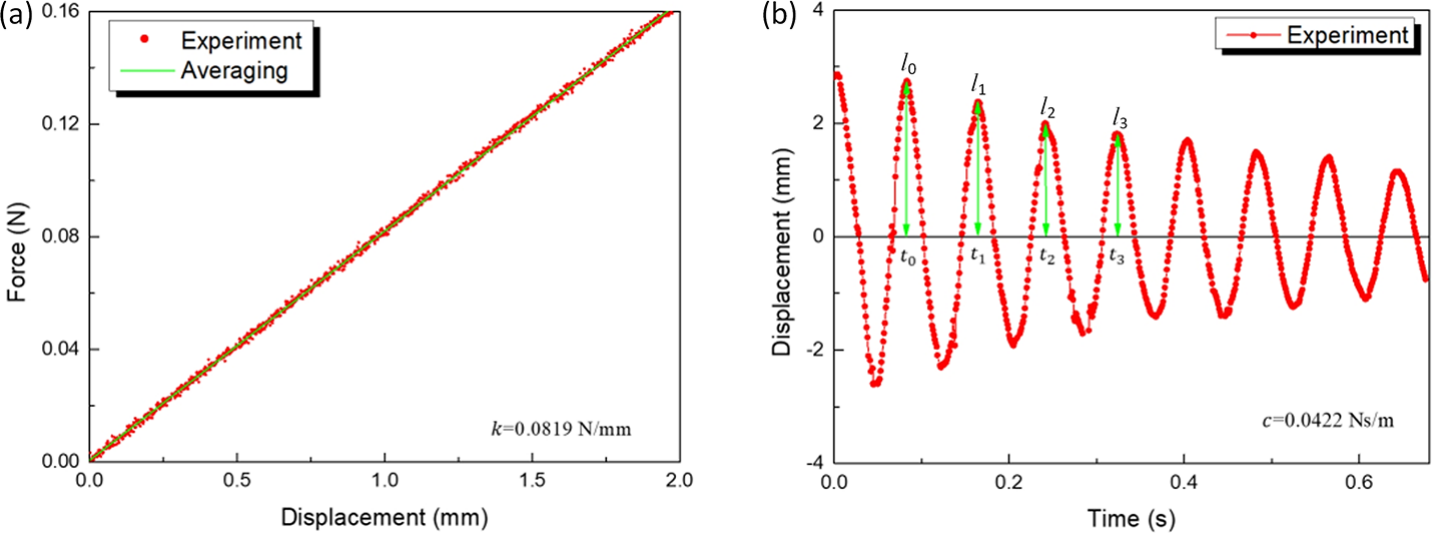
Characterization of the helical spring: (a) the force-displacement curve of static test of the helical spring indicating a stiffness coefficient of $k= 0.0819$ N/mm, (b) the free oscillation curve of the spring resulting in a damping coefficient of $c = 0.0422$ Ns/m

As the stiffness and damping coefficient of the helical spring are the main factors affecting the impact force generated by the magnet on the primary and secondary constraints of the capsule, the helical spring was characterized by using static compression experiments to obtain the force-deflection curve. Thus, the stiffness of the spring was measured to be 0.0819 N/mm, as shown in Fig. 5(a). For the damping coefficient of the spring, free oscillation experiments were conducted and the oscillatory displacement of the spring was detected by a laser displacement sensor as shown in Fig. 5(b). The damping coefficient can be described as

2 $$ c=2\sqrt{m_{v}k} \frac{\delta}{2\pi}, $$

where $m_{v}$ is the mass of the vibrating block, $\delta = (1/n) \ln (l_{0}/l_{n})$, $n=1, 2, 3, \ldots $ , and from Fig. 5(b), it can be calculated that the damping coefficient of the spring is 0.0422 Ns/m.

For the MBD modeling, the material properties of porcine colon tissue, the polyethylene capsule, and the helical spring were summarized in Table 1, including Young’s modulus $E_{int}$ [56], Poisson’s ratio $\nu_{int}$, and density $\rho _{int}$ of the large intestine, Young’s modulus $E_{cap}$, Poisson’s ratio $\nu_{cap}$, and density $\rho _{cap}$ of the capsule [26], spring stiffness coefficient $k$ and damping coefficient $c$. In addition, the friction coefficient $\mu $ between the capsule and the intestine is 0.2293.

**Table 1:** Material parameters of the capsule and the large intestine [26, 56]

Parameter	Unit	Value	Parameter	Unit	Value
$E_{int} $	MPa	0.01	$\rho_{int} $	kg/m^3^	1000
$E_{cap} $	MPa	1100	$\rho _{cap} $	kg/m^3^	1278
$\nu_{int} $	-	0.49	*c*	Ns/m	0.0422
$\nu_{cap} $	-	0.42	*k*	N/mm	0.0819
*μ*	-	0.2293			

### Determination of the magnetic actuation force

**Fig. 6:**
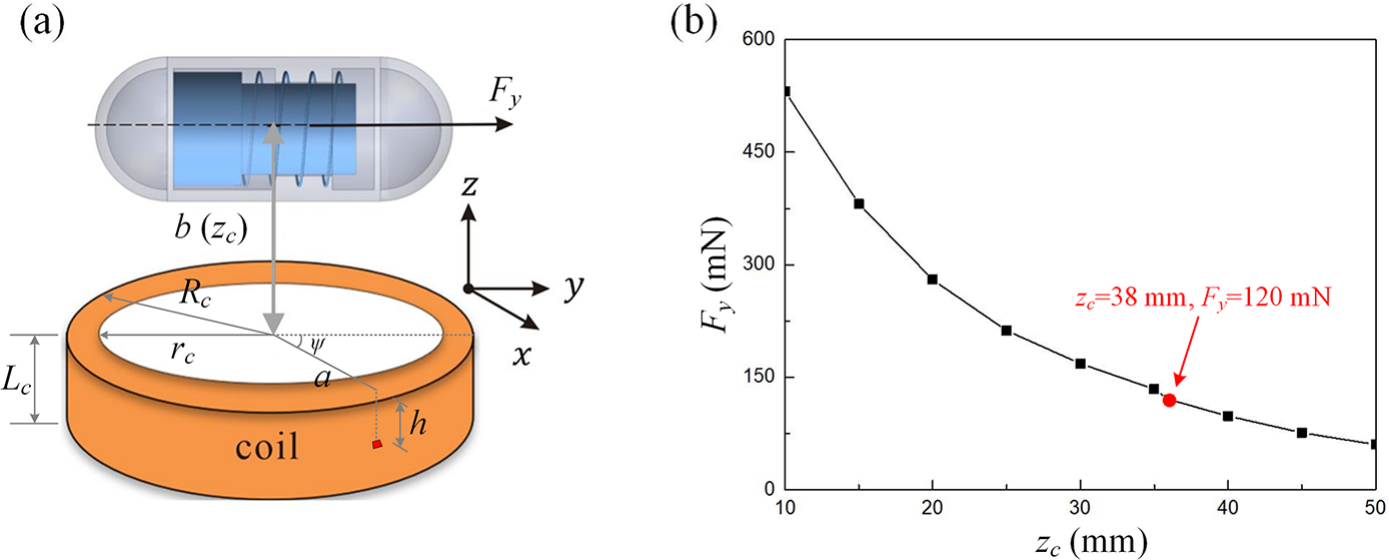
Characterization of the magnetic actuation force. (a) Schematic diagram of the relative position of the coil and the capsule, where the capsule is directly above the coil axis and is allowed to move along the $y$ axis under the magnetic field. (b) The calculation results of $F_{y}$ by using Eq. (3) and Eq. (4). the red point indicates the selected $z_{c}$ for later experiments, where $z_{c} = 38\text{ mm}$, $F_{c} = 120\text{ mN}$ (Color figure online)

For the propulsion of the capsule robot, an external magnetic field generated by a hand-held coil can remotely and precisely control the motion and position of the vibro-impact capsule. The coil is placed horizontally just below the capsule, with its central axis perpendicular to the capsule axis as shown in Fig. 6(a). Based on the previous optimization design of the hand-held coil [1], the upper capsule is mainly subject to the driving force in the horizontal direction, so the forces in the $x$ and $z$ directions are ignored. Thus, the horizontal driving force ($F_{y}$) on the capsule is depicted as

3 $$ \vec{F_{y}}=v \ (\vec{M}\cdot \nabla ) \ \vec{B}= v\ M \ { \frac{\partial B_{y}}{\partial x} } $$

and

4 $$ B_{y}=\frac{\mu _{0} I}{4\pi} \int _{0}^{L_{c}} \int _{r_{c}}^{R_{c}} \int _{0}^{2 \pi} \frac{a(b+h)\sin \psi}{\chi^{3/2}}\mathrm{d}\psi \mathrm{d}a \mathrm{d}h, $$

where $M$ is magnet’s magnetization value, $v$ is magnet’s volume, and $B$ is the magnetic field, $\mu _{0}$ is the magnetic constant, $I$ is the applied current to the coil, $L_{c}$ is the coil height, $R_{c}$ is the outer radius of the coil, and $r_{c}$ is the inner radius of the coil, $a$ is the radius of the coil wire element, $b$ is the vertical distance between the coil surface and the magnet’s center (i.e. $z_{c}$), $b+h$ is the vertical distance from the coil wire element to the magnet’s center, and $\chi= (x-a \cos \psi )^{2}+(y -a \sin \psi )^{2}+(b+h)^{2}$. Through calculations, the relationship between the driving force and the capsule-coil distance was found as shown in Fig. 6(b). For subsequent experiments, in order to keep a relatively far distance between the capsule and the coil to mimic the real operation while having enough force to actuate the capsule, $z_{c}$ was selected as 38 mm, and $F_{y}$ therefore is equal to 120 mN.

### Multibody dynamics modeling

Within the context of the MBD model, the interaction between the capsule and the intestinal wall primarily manifests as collisions, a phenomenon depicted in the software as follows [57]:

5 $$\begin{gathered} F_{\mathrm{impact}}= \textstyle\begin{cases} 0, \ q > q_{0}, \\ k_{c}(q_{0} - q)^{e} - C_{\mathrm{max}}\cdot (d_{q}/d_{t})\cdot \mathrm{STEP}(q,q_{0},-d,1,q_{0},0), \ q\leq q_{0}, \end{cases}\displaystyle \end{gathered}$$

6 $$\begin{gathered} k_{c}=\tfrac{4}{3}\sqrt{R^{*}} E^{*}, \end{gathered}$$

7 $$\begin{gathered} E^{*} =(\tfrac{1-\nu_{int}^{2}}{E_{int}}+\tfrac{1-\nu_{cap}^{2}}{E_{cap}})^{-1}, \end{gathered}$$

8 $$\begin{gathered} R^{*} =(\tfrac{1}{R}+\tfrac{1}{R_{cap}})^{-1}, \end{gathered}$$

where $F_{\mathrm{impact}}$ is a function of the instantaneous collision contact force between the capsule and the intestinal wall, $E^{\ast}$ and $R^{\ast}$ represent the equivalent radius and the equivalent Young’s modulus, respectively. Among the parameters, $k_{c}$ represents the stiffness coefficient of the collision between the capsule and the intestine, and $R_{cap}$ denotes the radius of the capsule. The initial separation distance between the two objects prior to collision is $q_{0}$, while $q$ denotes the actual distance between the objects during the collision. The rate of change in this distance over time, or velocity, is expressed as $d_{q}/d_{t}$. Additionally, $C_{max}$ indicates the maximum damping coefficient, and $d$ represents the depth of penetration, which is critical in determining the point at which the damping force peaks. The STEP function is a built-in function in the MSC Adams software. It is used to apply a continuous load by entering a time point and the magnitude of the force. In the software, its basic expression is STEP ($x$, $x_{0}$, $h_{0}$, $x_{1}$, $h_{1}$), which can be expanded to read as

9 $$ \mathrm{STEP}= \textstyle\begin{cases} h_{0}, x \leq x_{0}, \\ h_{0}+\kappa \cdot \Delta ^{2}(3-2\Delta ), x_{0} < x < x_{1}, \\ h_{1}, x \geq x_{1}, \end{cases} $$

where $\kappa=h_{1}-h_{0}$, $\Delta=(x-x_{0})/(x_{1}-x_{0})$, and $x$ is the independent variable, which can be time or any function of time. In this model, it is used as the time of a single cycle of excitation to adjust the frequency of the driving force. $x_{0}$ is the starting value of the STEP function of the independent variable, that is, time when the driving force is 0 mN. $h_{0}$ is the initial value of the STEP function, and the input value is 0. $x_{1}$ is the end value of the STEP function of the independent variable, that is, the time point when the driving force becomes 120 mN and continues until the end of the cycle. $h_{1}$ is the final value of the STEP function, which is 0.12 at this time. Based on the STEP function, the results can be automatically repeated, thereby achieving a continuous periodic square wave-type driving force. For example, a driving force with an amplitude of 120 mN, a frequency of 50 Hz, and a duty cycle of 0.2 can be written as STEP$\{\mathrm{mod} (\mathrm{time}, 0.02), 0.0159, 0, 0.016, 0.12\}$ in the STEP function. In the function, $10^{-4}$ second was used for amplitude variation to achieve a square wave signal.

**Fig. 7:**
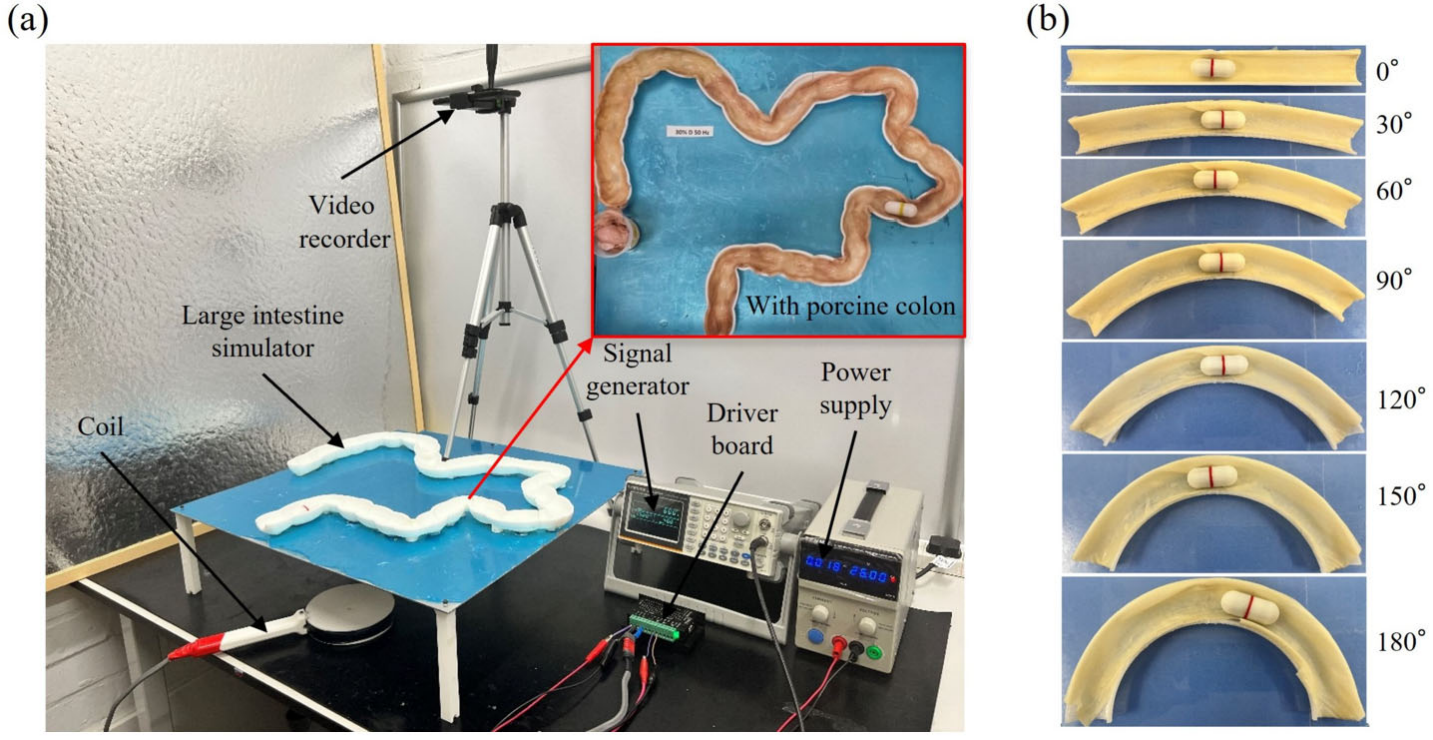
Photograph of the experimental setup. (a) The capsule passes through the entire large intestine from the rectum to the cecum. A signal generator generates periodic square wave signals on the coil at different frequencies and duty cycles through a driver board driven by a power supply. A top video recorder records the movement trajectory of the capsule. (b) The capsule robot moves on the semi-tubular intestines with different bending radii, ranging from 0^∘^ to 180^∘^, with an interval of 30^∘^. The excitation frequency and the duty cycle of the capsule robot are fixed at 60 Hz and 0.4, respectively

In the MBD simulation model, the large intestine wall in the experiment with Young’s modulus of only 0.01 MPa. Therefore, in the MBD model, it is defined as a flexible body with a 2 mm mesh size. To ensure that the large intestine is flexible and does not undergo significant deformation during the process of passing through the capsule, the outermost layer of the flexible intestine is designed with a rigid support with the same structural shape, which is consistent with the large intestine simulator in Fig. 7(a). To accurately simulate the interaction between capsules and the intestinal environment, a hybrid modeling method was adopted. Previous studies have shown that when magnets are subjected to periodic constraints, the capsule shell undergoes slight deformation. To be closer to the motion state of the capsule in the actual experimental environment, the capsule shell is also defined as a flexible body in this model. Therefore, finite element software ANSYS was used to conduct flexible body modeling of the intestine and the capsule shell to capture their elastic properties. Input Young’s modulus, Poisson’s ratio, and density corresponding to the intestine and the capsule in Table 1 and use the tetrahedral three-dimensional solid element solid 45, which is suitable for complex geometric shapes. In addition, the entrance surface of the large intestine and the load-bearing structure in the capsule were designed as rigid regions, and these two flexible bodies were output to MSC Adams software for MBD simulation. In MSC Adams, the internal magnet of the capsule was modeled as a rigid body. Based on the flexible large intestine model, a rigid body support with a completely identical shape was constructed on its outermost layer to maintain consistency with the experimental setup. The magnetic driving force and the contact interaction between the capsule and the intestinal wall are integrated into the simulation, allowing for coupled dynamic analysis. Based on computational efficiency and accuracy, the flexible mesh size for the intestine and the capsule shell is selected as 0.5 mm. This value ensures a balance between computational cost and numerical accuracy, as finer grids generate diminishing returns on accuracy, while coarser grids introduce significant bias. The selected grid size can accurately represent the deformation of the intestinal wall and the interaction with the capsule. Furthermore, the following assumptions were made in our simulation:

- The intestine is isotropic and homogeneous.
- The capsule shell is made of linear elastic material.
- The inner magnet and the large intestine’s support holder are rigid bodies.
- The inner magnet performs frictionless motion via the linear bearing of the capsule.
- The contact between the large intestine wall and its support holder is fixed.

In the MBD simulation conducted using MSC Adams, the wall thickness of the porcine large intestine layer was set as 1.5 mm, and it was positioned flat on a fixed holder at the bottom. The software simplifies the spiral spring between the magnet inside the capsule and the capsule shell as a virtual spring, with a strength coefficient of $k = 0.0819\text{ N}/\text{mm}$ and a damping coefficient of $c = 0.0422\text{ Ns}/\text{m}$ as shown in Table 1. The driving force acting on the magnet in the model is a periodic square wave signal with an amplitude of 120 mN. The change in the square wave driving force is controlled by the insertion time function based on the response time of the corresponding frequency and duty cycle. The direction of the driving force is fixed on the axis of the magnet and always perpendicular to the surface of the magnet. The friction coefficient $\mu $ between the capsule and the intestinal wall is 0.2293. The gravity field is also loaded into the model. In each model, the capsule was set to travel from the rectum to the cecum.

The MBD visualization platform was configured within MSC Adams to simulate and analyze the interactions between the capsule and the intestinal environment. While the visualizations generated by Adams provide valuable insights into capsule dynamics, the platform was specifically tailored for this study to model complex anatomical structures, optimize mesh configurations, and accurately parameterize contact forces and driving mechanisms. These visualizations complement the quantitative analysis of key metrics, such as velocity profiles, resistance forces, and passage times, which are used to validate the model and guide experimental design.

## Experimental setup and procedure

Similar to the MBD simulation, the vibro-impact capsule consists primarily of a capsule shell, internal permanent magnets, and helical springs used in the experimental investigations. The external hand-held coil is positioned at the bottom of the capsule as illustrated in Fig. 7(a). Electromagnetic principles are utilized to ascertain the forces exerted on the internal magnets within the capsule. A phantom of the human colon, incorporating its flexures, folds, and irregular surfaces, was constructed based on anatomical data. This model ensures accurate simulation of the large intestine’s anatomical structure, thereby allowing experiments to be conducted in a setting that closely approximates real-life conditions. Furthermore, an additional experiment was devised to effectively assess the capsule’s guidance capabilities. This involved monitoring the capsule’s movement through segments of the large intestine with varying bending radii as shown in Fig. 7(b). This experiment provides insight into how the capsule navigates and adapts to varying degrees of flexure, an essential factor in assessing its overall performance. Also, both the experimental and simulation setups utilized a half-open intestine model to simplify observation and focus on the primary dynamics of the capsule’s motion. In the simulation, the half-open intestine included flexible boundaries to represent the mechanical properties of the intestinal wall, ensuring consistency with the experimental model. This approach maintains relevance to real intestinal conditions as the primary mechanical interactions, such as resistance, capsule speed, and propulsion dynamics, occur at the capsule-intestinal wall interface, which is preserved in the half-open model.

### Capsule movement in the entire large intestine with varying excitation parameters

Figure 7(a) illustrates the experimental setup of a capsule moving in the entire large intestine. The capsule is actuated by the magnetic field generated by a hand-held coil below. The capsule shell is fabricated from 3D-printed polyethylene, featuring a wall thickness of 1.5 mm and a mass of 5.98 g. Within the capsule, a T-shaped magnet composed of neodymium iron boron (NdFeB) weighing 24.63 g is housed. The signal generator produces a periodic square wave signal, which is then amplified and transmitted to the coil. The signal generator is capable of modulating various frequencies and duty cycles in the coil. The overall movement of the capsule within the intestinal tract is recorded by a top-mounted camera. A porcine intestine is supported by a holder with a similar structure to preserve its shape. The recorded footage is analyzed to determine the state and velocity of the capsule. In order to identify the optimal excitation parameters for its complete movement through the large intestine, the capsule was controlled using frequencies between 30 Hz and 60 Hz and duty cycles from 0.1 to 0.5.

### Capsule movement in colon segments with varying bending angles

In order to study the effect of different angles of colon curvature on the capsule movement speed, the capsule was driven in the experimental model as shown in Fig. 7(b). The intestinal segment curvature angle varies from 0^∘^ to 180^∘^ with an interval of 30^∘^. The length of each intestinal segment model is consistent, which is 300 mm. In each experiment, the capsule moves from left to right. The square wave signal generated by the coil is always maintained at an amplitude of 120 mN, a frequency of 60 Hz, and a duty cycle of 0.4.

## Experimental and MBD results

This section focuses on describing the effective evaluation of the accuracy of the MBD model by comparing the results generated by actual experiments and MBD simulations in both Scenarios 1 and 2. Combining real experiments with simulations provides a more complete understanding of how the vibro-impact capsules interact with the large intestine. The results of Scenario 3 demonstrate the capsule’s ability to move over a colonic segment of varying dimensions, clearly demonstrating the main dimensional factors that influence capsule motion to optimize the design of the capsule’s structure and dimensions. The comparison of the results of these three scenarios can also be viewed more intuitively by the supplementary video.

### Capsule excitation variation in the entire large intestine: Scenario 1

**Fig. 8:**
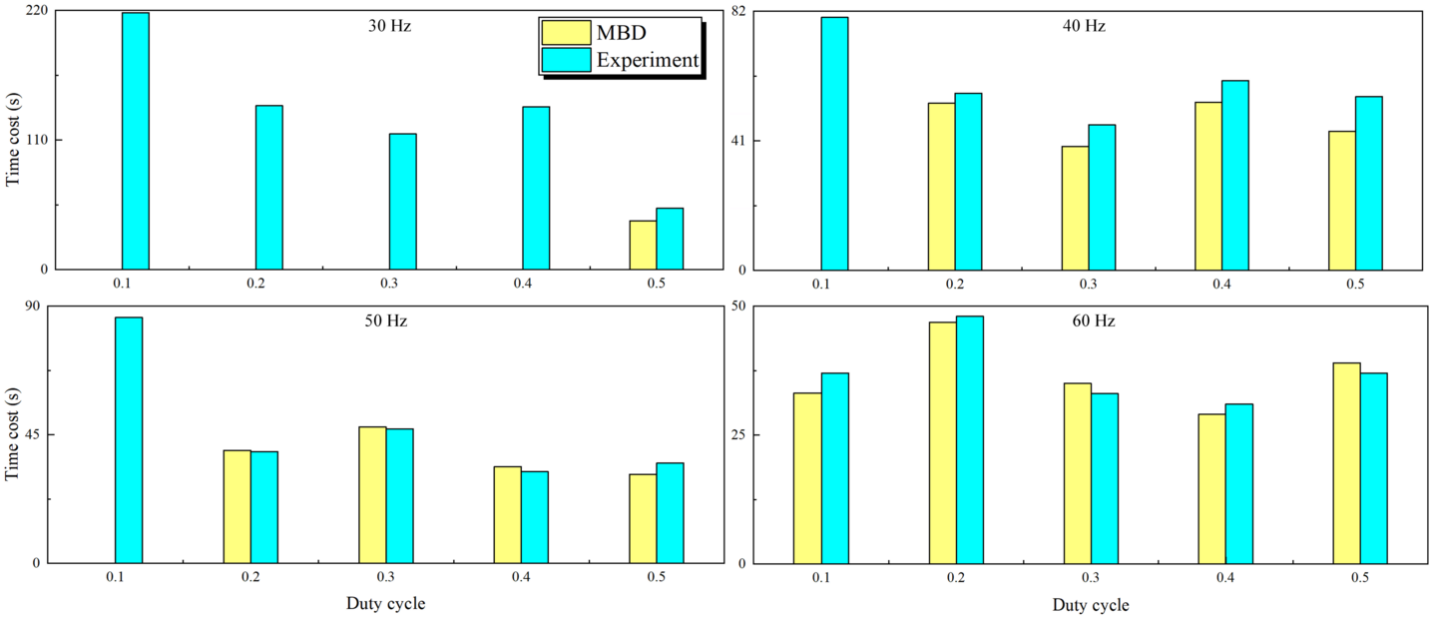
Comparison of the times taken for capsule movement throughout the entire large intestine by experiments and MBD simulations. The capsule robots pass through the large intestine at the excitation frequencies of 30 Hz, 40 Hz, 50 Hz, and 60 Hz with a duty cycle of 0.1 to 0.5 and a fixed amplitude of 120 mN. The yellow bars represent the MBD simulation results, and the cyan bars represent the experimental results (Color figure online)

In Scenario 1, the results of experiments and MBD simulations with variations in excitation parameters are compared. Figure 8 depicts the time taken by the capsule to pass through a large intestine of 1.6 meters in length for an excitation amplitude of 120 mN but with different frequencies and duty cycles. The difference between the multibody simulations and the actual experimental results is not significant. However, the capsule failed to traverse the entire large intestine model correctly in the MBD simulations for the following conditions: a frequency of 30 Hz with duty cycles ranging from 0.1 to 0.4, a frequency of 40 Hz with a duty cycle of 0.1, and a frequency of 50 Hz with a duty cycle of 0.1. Furthermore, both actual and MBD simulations indicate that using high frequency with a high-duty cycle prolongs the capsule’s passage time through the large intestine, suggesting that the optimal duty cycle varies with different frequencies. Experimental and simulation results demonstrate that with the excitation parameter set at a frequency of 60 Hz and a duty cycle of 0.4, the capsule achieves the shortest passage time, recorded at 31 seconds and 28.2 seconds, respectively.

**Fig. 9:**
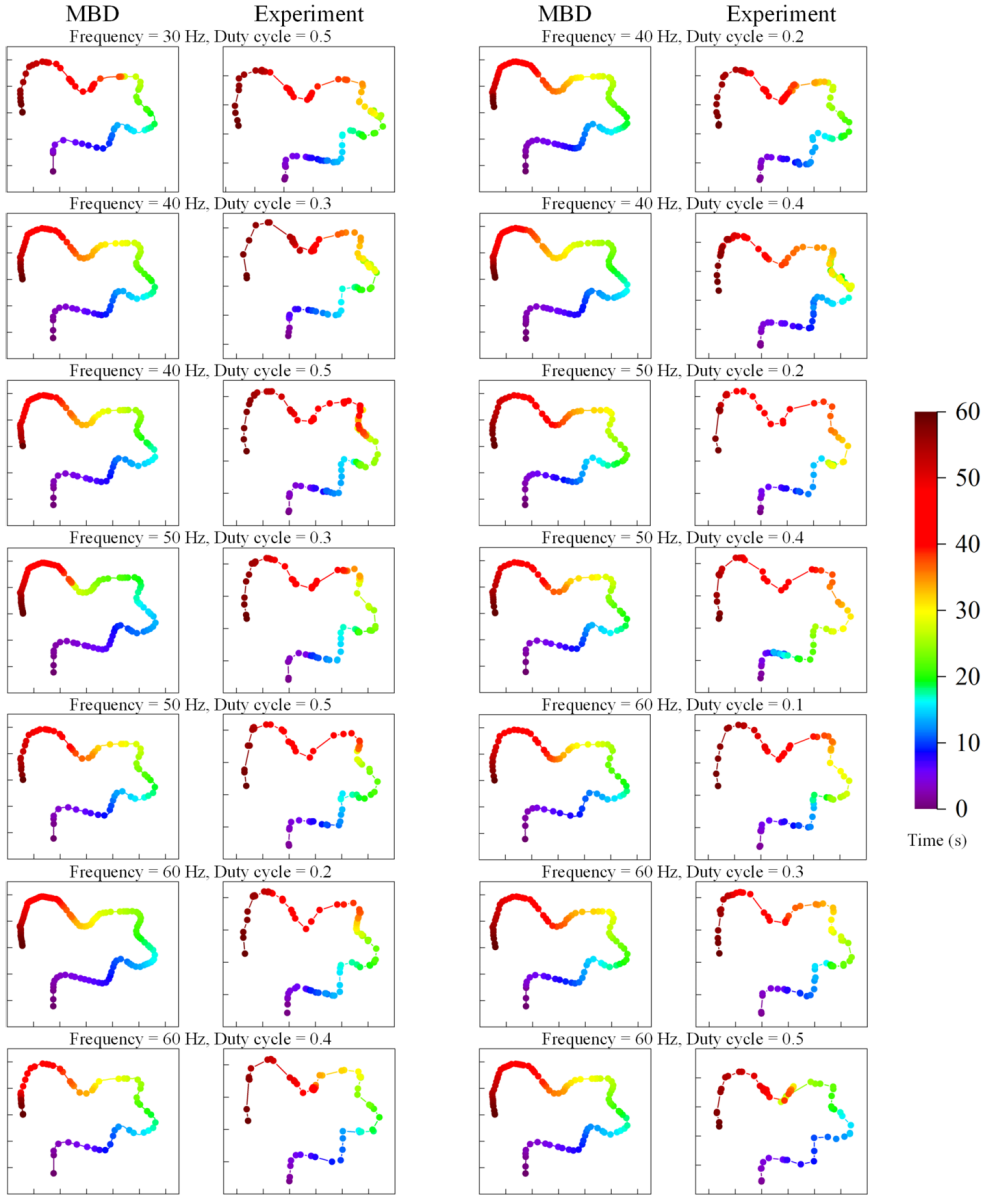
The trace plots of MBD and experimental results with two sets of result comparison charts in each row, each set of comparison plots containing MBD results (left) and experimental results (right), arranged in the order of 30 Hz, 40 Hz, 50 Hz, and 60 Hz, and corresponding frequencies and duty cycles marked at the top of each comparison plot. The color bar on the right panel indicates the travel time (Color figure online)

Figure 9 provides a comprehensive comparison of the motion paths (trace plots) of a vibro-impact capsule in a complete large intestine model under various frequencies and duty cycles, obtained from MBD simulations and experimental results. Overall, the MBD simulations closely replicate the experimental traces, particularly in terms of path shapes and time distributions, demonstrating the reliability of the computational model. The results reveal that higher frequencies (50 Hz and 60 Hz) and duty cycles (0.4 and 0.5) significantly enhance the capsule’s propulsion, leading to faster and longer motion paths. In contrast, lower frequencies (30 Hz and 40 Hz) and duty cycles (0.2 and 0.3) result in slower and shorter traces, as insufficient excitation prevents the capsule from overcoming resistance. Both simulation and experimental results highlight the nonlinear relationship between excitation parameters and motion efficiency, underlining the importance of parameter optimization for improved capsule performance.

Compared to the experimental results, the capsule’s motion in the MBD trace plots appears smoother with minimal backward movement, as also demonstrated in the accompanying comparative supplementary video. This discrepancy arises primarily due to the experimental setup, where the capsule was driven by a hand-held energized coil, resulting in variations in the driving force’s magnitude and direction. Additionally, in practice, the magnetic force acting on the capsule’s internal magnet is a three-dimensional force. Aside from the axial component $F_{y}$, the magnet also experiences minor lateral and vertical forces, as previously detailed in our work [1]. However, these lateral and vertical forces are negligible compared to $F_{y}$ and the specified distance between the magnet and the coil in the experiment has a limited impact on the capsule’s overall motion. To optimize computational efficiency, the MBD simulation applies a consistent unidirectional axial force to the capsule’s internal magnet, ensuring stable forward motion. These factors result in the MBD simulations exhibiting more stable motion, while the experimental traces frequently display repeated backward movements due to the instability in the experimental driving condition.

**Fig. 10:**
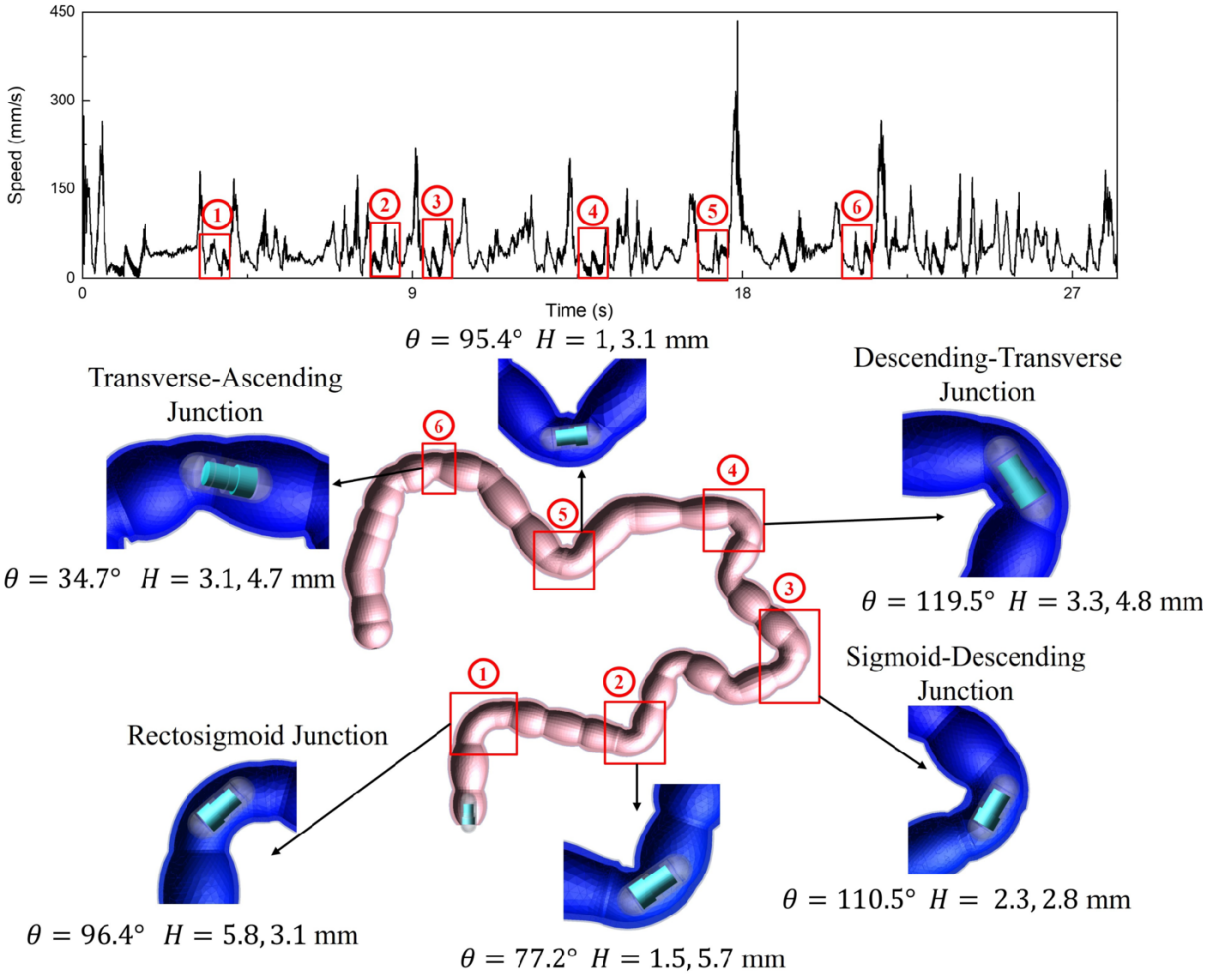
Time history of speed of the capsule robot moving through the entire large intestine, and the snapshots of the MBD simulation at where the robot has low speeds. The capsule driving frequency was 60 Hz, the duty cycle was 0.4, and the amplitude was 120 mN. The low-speed area is marked by a red box in the time history and labeled with a number from 1 to 6. The snapshots below show the specific positions of the capsule in the six low-speed movement zones, including large flexures ($\theta$), high folds ($H$), rectosigmoid junction, sigmoid-descending junction, descending-transverse junction, and transverse-ascending junction. Each snapshot shows the heights of two folds, one in front of and one behind the capsule (Color figure online)

The top plot of Fig. 10 shows the velocity time course of the capsule in the MBD simulation at the optimal excitation parameter, from which it can be seen that the capsule has six low-velocity zones during its movement, which correspond to the junctions of the large intestine model (the bottom plot of Fig. 7). The presence of higher folds ($H$) at these junctions causes the capsule to lift its head into a crossing state, and the bending of the junctions ($\theta$) causes the capsule to collide into a turned state. These features change the motion attitude of the capsule while causing the capsule to move at a rapidly decreasing speed.

Lower excitation parameters in the simulation lead to shorter excitation times, which makes it impossible for the capsule to obtain sufficient driving force to move forward. In addition, the error between the MBD simulation results and the actual experimental results is because, in the actual experiments, the magnet inside the capsule is driven by the electromagnetic force in a three-dimensional space, and because the coil is hand-held, which leads to a difficulty in stably controlling the distance and direction between the coil and the capsule. However, by comparing the results of the changes in the excitation parameters, the MBD simulation can accurately reflect the physical behavior of the vibro-impact capsule in the large intestine with less error, although it cannot completely restore the actual experiment. This makes MBD modeling an essential tool for analyzing and simulating the motion of the capsule performed in the intestine. Moreover, by studying the results of the fastest motion velocity, it was found that the curved and folded structures in the intestine significantly influence the motion tendency of the capsule.

### Bending angle variation in large intestine segments: Scenario 2

**Fig. 11:**
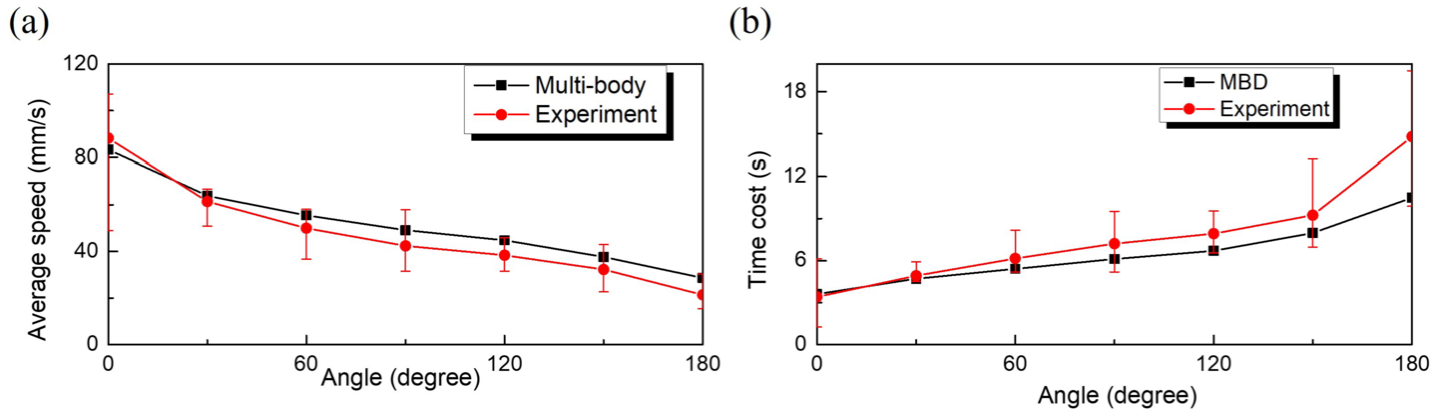
Capsule’s average speed (a) and transit time (b) in different curved intestinal segments under a frequency of 60 Hz, a duty cycle of 0.4 and an amplitude of 120 mN. The black line represents the MBD results, while the red line represents the experimental results (Color figure online)

In Scenario 2, MBD simulations and experiments were performed on seven colon segments with different flexure angles. As illustrated in Fig. 11(a), the experiments show that the average speed of the capsule decreases from 88.23 mm/s to 20.27 mm/s with an increasing deflection angle when subjected to a driving force of 120 mN, a frequency of 60 Hz, and a duty cycle of 0.4. Similarly, in the MBD simulations, the average velocity decreased from 83.33 mm/s to 28.57 mm/s. This reduction in velocity is attributed to the fact that the magnetic force in the vibro-impact capsule is primarily axial. Consequently, the head of the capsule impacts the intestinal wall, leading to a decrease in velocity with increasing deflection angles. In addition, the capsule navigates and steers by colliding its head with the intestinal wall, enabling movement even at a 180^∘^ deflection angle. This indicates that the capsule can pass through the colon intact through magnetic control. Furthermore, the MBD simulation results exhibited less than a 40.9% deviation from the actual maximum, highlighting its accuracy.

In Fig. 11(b), the time spent by the capsule in the experiment and simulation increases from 3.4 s to 14.8 s and from 3.6 s to 10.5 s, respectively. It can be seen from this pair of graphs that when the angle of the intestine is greater than 30^∘^, the results between the simulation and the experiment begin to show a certain degree of error, which is more obvious at an angle of 180^∘^. This phenomenon confirms that the capsule has some steering and navigational ability under MBD simulation, relying only on the axial driving force. On the other hand, it also reconfirms that controlling the steering of the capsule utilizing a hand-held external coil, especially when the turning angle is too large, is not a completely stable way compared to the simulation experiments in the ideal state. These findings highlight challenges in designing external coils for more stable maneuvering and enhancing the capsule’s steering and navigation capabilities. The propulsion efficiency and maneuverability of the capsule were evaluated under varying excitation conditions, with performance indicators including capsule speed, trajectory accuracy, and energy consumption. These metrics are critical for assessing the system’s readiness for clinical application.

### Structural size variation in large intestine segments: Scenario 3

**Fig. 12:**
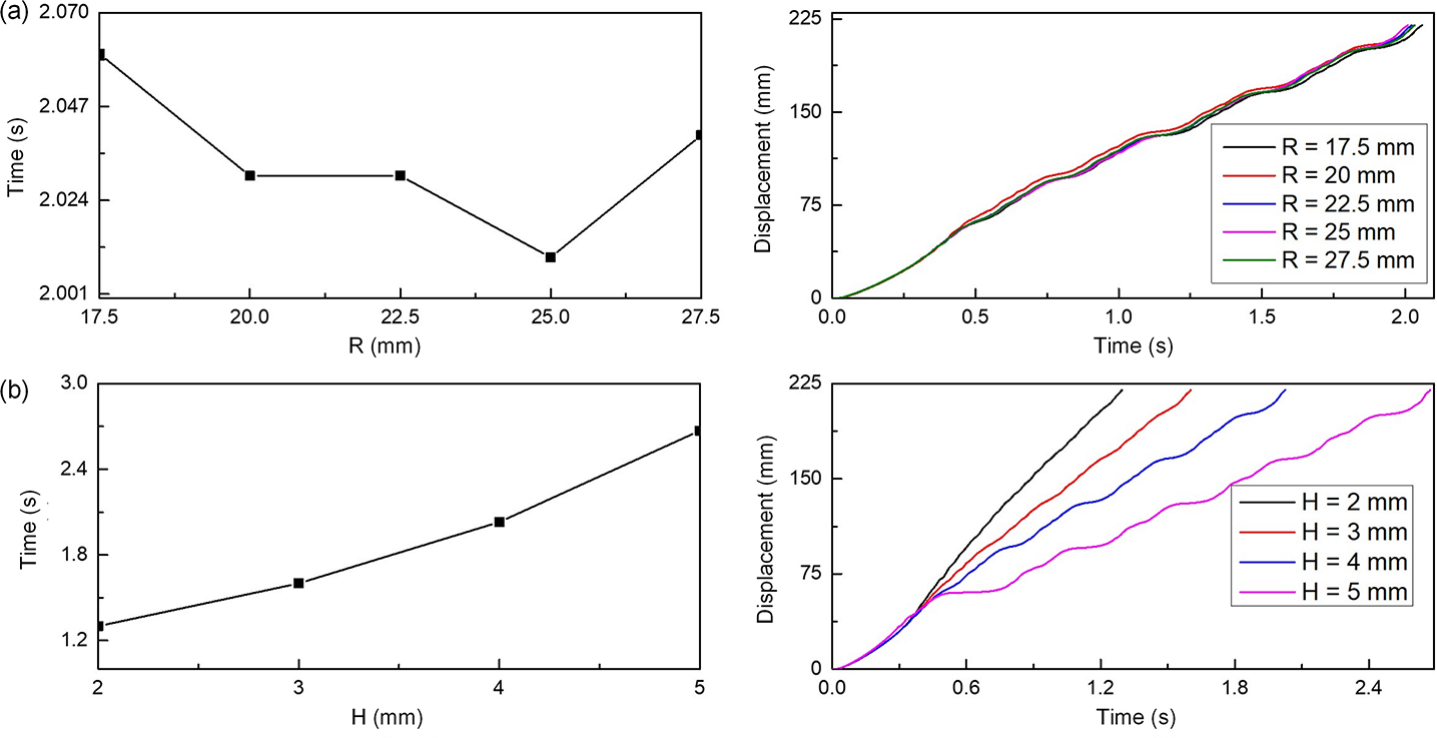
Time cost and time history results of capsule movement in the intestine with different intestinal radii (a) and folding heights (b) at a frequency of 60 Hz, a duty cycle of 0.4, and an amplitude of 120 mN. The radius of the intestine changes from 17.5 mm to 27.5 mm with 2.5 mm intervals and the folding height changes from 2 mm to 5 mm with 1 mm intervals (Color figure online)

For Scenario 3 in the MBD model, capsule motion was analyzed across varying haustral lengths ($L$), radii ($R$), and folding heights ($H$) of colon segments. Figure 12 illustrates the motion time of the capsule in response to the changes in radius and fold heights under excitation parameters of 120 mN force, 60 Hz frequency, and a 0.4 duty cycle. Figure 12(a) demonstrates that when the haustral radius varies from 17.5 mm to 27.5 mm, the movement time remains nearly constant with only a 0.05 s difference between the shortest and longest times. The horizontal displacement history further reveals that the movement trend and displacement change of the capsule are nearly identical across different radii. This suggests that when the capsule’s radius approaches or exceeds the haustral radius, the effect on its progression is minimal. Figure 12(b) shows that increasing the folding height from 2 mm to 5 mm lengthens the capsule’s movement time from 1.3 s to 2.67 s. However, at a height of 6 mm, the capsule fails to fold over successfully. The displacement history indicates that with greater folding height, the capsule’s duration on the folding structure increases, implying a higher requirement for the capsule’s crossing capability.

**Fig. 13:**
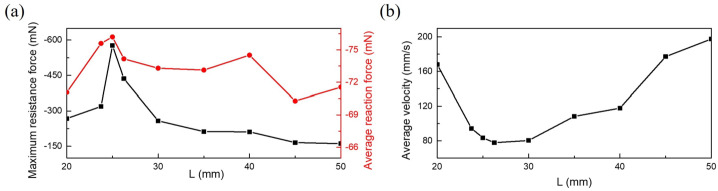
The influence of intestinal haustral lengths on the capsule motion resistance force (a) and average velocity of the capsule (b). The black line shows the maximum resistance force and red line shows average resistance force in the horizontal direction when the capsule moves (Color figure online)

As illustrated in Fig. 13, in the horizontal orientation, alterations in haustral length from 20 mm to 25 mm yield an escalation in maximum resistance from −268.18 mN to −576.95 mN, subsequently dwindling to −161.27 mN as the length extends to 50 mm. Nonetheless, the peak average reaction force remains at 25 mm, recorded as −76.2 mN, while the lowest point occurs at 45 mm with a value of −70.29 mN. Moreover, considering average velocity, the trough emerges at 77.83 mm/s for a length of 26.25 mm, contrasting with the highest speed of 197.6 mm/s observed at a length of 50 mm. This underscores that resistance is merely one determinant impacting capsule motion across varying haustral lengths; the capsule’s motion posture also exerts a significant influence on speed.

**Fig. 14:**
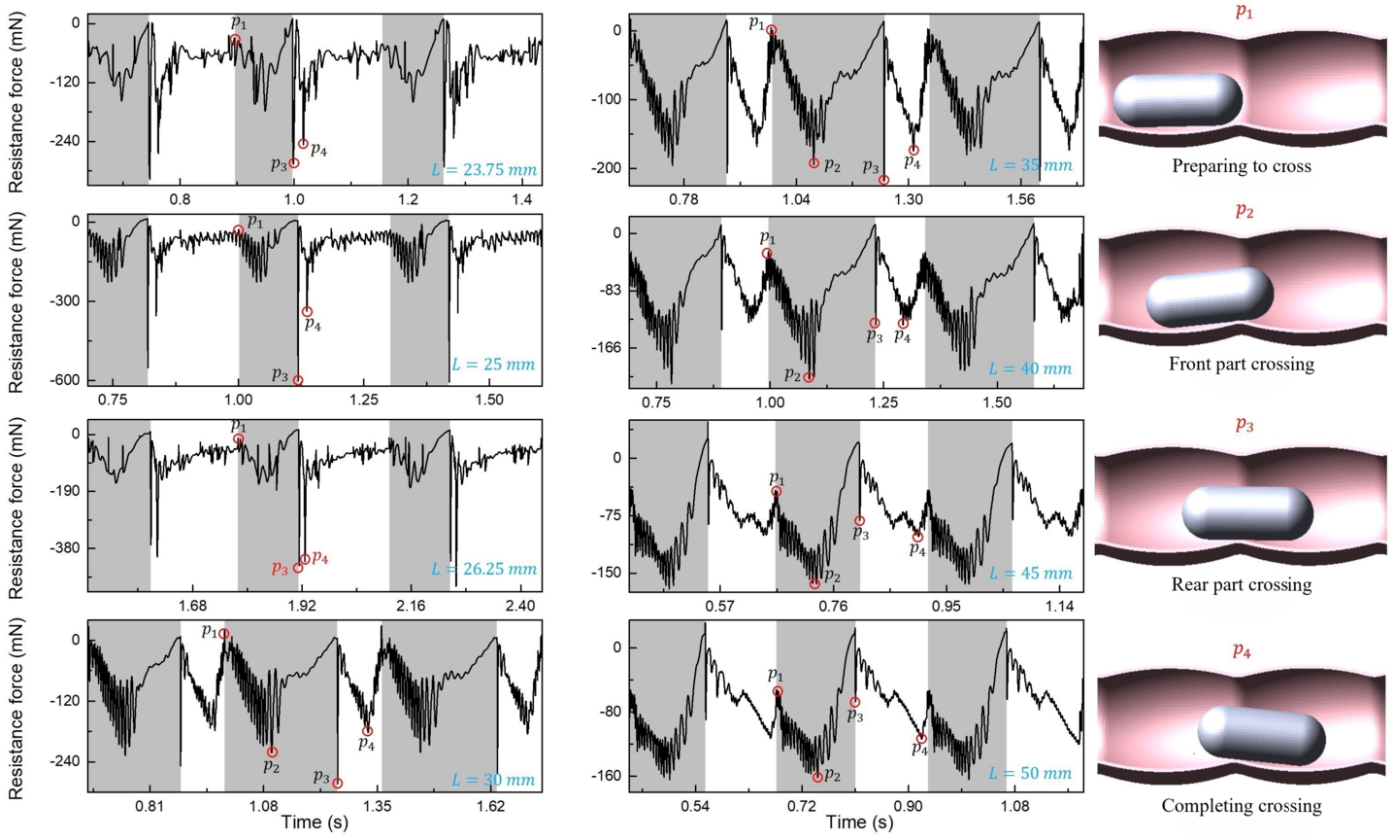
The four resistance periods from top to bottom on the left side are 23.75 mm, 25 mm, 26.25 mm, and 30 mm, respectively. On the right side, from top to bottom they are 35 mm, 40 mm, 45 mm, and 50 mm, respectively. The grey area represents the time it takes for the front part of capsule to cross folds, while the white area represents the time it takes for the rear part of capsule to cross folds. The four typical positions of the capsule in the crossing action are marked with red circles on the far right. The typical posture for capsule crossing is to prepare to cross ($p_{1} $), the front part of capsule crossing ($p_{2} $), rear part crossing ($p_{3} $), and completing crossing ($p_{4} $)

Figure 14 shows the resistance history of the capsule when the length of the intestine changes through MBD simulation. The grey and white areas represent the time zones during which the front and the tail of the capsule crosses the folding structure, respectively. The order from top to bottom is 23.75 mm, 25 mm, 26.25 mm, 30 mm, and 35 mm, 40 mm, 45 mm, and 50 mm. As can be seen from Fig. 14, four primary stages observed during the capsule’s folding movement cycle are presented, where red circles denote the initiation of crossing ($p_{1}$), the front-end crossing ($p_{2}$), the back-end crossing ($p_{3}$), and the completion of crossing ($p_{4}$). In the length range of $L\in [23.75, 26.25]\text{ mm}$, the duration of front-end crossing is relatively brief, with notable resistance concentrated at action points $p_{3}$ and $p_{4}$.

This phenomenon arises due to the capsule’s insufficient length, resulting in a shortened time interval between $p_{3}$ and $p_{4}$. Gravity also exerts an influence on the capsule, while the folding structure imparts substantial resistance during the crossing process. This deduction is further supported by lengthening the capsule to 30 mm, where $p_{2}$ encounters significant resistance, leading to a higher proportion of time dedicated to front-end crossing within the cycle.

In addition, the duration of the tail crossing in the cycle gradually increases in the length range of 35 mm to 50 mm, with $p_{2}$ becoming the location with the largest resistance during the crossing process. It is noteworthy that the resistance at $p_{3}$ experiences a swift decline, while the temporal gap between $p_{4}$ and $p_{1}$ steadily contracts. This observation confirms that when the capsule length is sufficiently extended or exceeds its traversal length, the front section of the capsule will undergo accelerated crossing, which helps to quickly pass through the capsule.

In summary, through the MBD simulation results, it can be found that when the intestine radius is about twice that of the capsule, the movement speed of the capsule will not be affected, as the contact area between the capsule and the intestinal wall will not change with the increase in intestine radius. In addition, the results of the change in folding height show that the higher the fold, the higher the requirement for the climbing ability of the capsule. When the folding height exceeds 30% of the capsule diameter, the vibro-impact capsule cannot complete the climbing. On the other hand, the most significant factor affecting the movement of capsules with different lengths of haustral is the change in resistance during the crossing action. When the length is close to half the length of the capsule, the folding structure will generate higher resistance and slow down the crossing speed. However, when the length is longer or even exceeds the length of the capsule, although the decrease in resistance is not significant, the area with higher resistance will complete the crossing at the front end. The crossing motion gradually becomes smoother and advances faster.

## Conclusions

This article studied the motion of a vibro-impact capsule robot within the large intestine using multibody dynamics (MBD) modeling and experimental investigation. The study considered three scenarios of capsule movement based on the anatomical structure of the large intestine. Initial experiments validated the MBD model’s accuracy, analyzing colon segment deflection changes and the influence of intestinal geometry on robot’s movement. The MBD model’s simulation of the capsule’s contact motion in the large intestine matches well with experimental results, demonstrating the capsule’s navigation and climbing capabilities. However, the MBD model struggles to simulate low frequency and low duty-cycle excitations accurately.

The optimal parameters for capsule motion were found to be a frequency of 60 Hz and a duty cycle of 0.4. The intestinal junction area is characterized by strong deflections and high folding, which significantly affects the capsule’s motion speed, so the operator needs to focus on these areas during control. Time costs for a complete pass through in experimental results slightly exceeded those of the MBD model, especially with larger bending angles, such as 180^∘^, where significant variations occurred due to the manual navigation of the capsule robot. Capsule movement speed decreased substantially with increased bending angles by 34.5% and 47.3% for 120^∘^ to 180^∘^ deflections in simulations and experiments, respectively.

In addition, capsule movement was influenced by geometric factors such as intestinal radius, intestinal length, and folding height. When the intestinal radius is close to or greater than its diameter, movement speed and posture remain unaffected. However, a folding height exceeding $3/5$ of the capsule radius reduces its overtaking capability. The capsule encounters maximum resistance when the haustral length is near its length, requiring more time for movement. When the haustral length exceeds the capsule’s length, movement becomes smoother and faster.

In conclusion, in this study, the results of these three scenarios demonstrate that the MBD model can reproduce the actual experiments for the capsule robot moving on the porcine large intestine, revealing the dynamic effects of different anatomies of the large intestine on the robot. Simulating MBD can improve research efficiency and save research costs. In addition, future capsule designs can explore streamlined shapes to reduce resistance, textured surfaces to enhance wall deflection, and capsule shells of different sizes to more effectively navigate complex intestinal geometries.

## Supplementary Information

Below is the link to the electronic supplementary material.

(MP4 4.9 MB)

## Data Availability

The computational and experimental data sets generated and analysed during the current study are available from the corresponding author on reasonable request. Example data has been provided as a video clip within the supplementary information file.
